# Iron Oxide/Polymer Core–Shell Nanomaterials with Star-like Behavior

**DOI:** 10.3390/nano11092453

**Published:** 2021-09-21

**Authors:** Virginie Vergnat, Benoît Heinrich, Michel Rawiso, René Muller, Geneviève Pourroy, Patrick Masson

**Affiliations:** 1 Institut de Physique et Chimie des Matériaux de Strasbourg (IPCMS), CNRS, Université de Strasbourg, UMR7504, 23 Rue du Lœss, BP 43, 67034 Strasbourg, France; virginie.vergnat@ipcms.unistra.fr (V.V.); genevieve.pourroy@ipcms.unistra.fr (G.P.); 2 Institut Charles Sadron (ICS), CNRS, Université de Strasbourg, UPR 22, 23 Rue du Lœss, BP 84047, 67034 Strasbourg, France; michel.rawiso@ics-cnrs.unistra.fr (M.R.); rene.muller@ics-cnrs.unistra.fr (R.M.)

**Keywords:** hybrid materials, non-aggregated nanoparticles, grafting from method, X-ray scattering, neutron scattering, star polymers

## Abstract

Embedding nanoparticles (NPs) with organic shells is a way to control their aggregation behavior. Using polymers allows reaching relatively high shell thicknesses but suffers from the difficulty of obtaining regular hybrid objects at gram scale. Here, we describe a three-step synthesis in which multi-gram NP batches are first obtained by thermal decomposition, prior to their covalent grafting by an atom transfer radical polymerization (ATRP) initiator and to the controlled growing of the polymer shell. Specifically, non-aggregated iron oxide NPs with a core principally composed of γ-Fe_2_O_3_ (maghemite) and either polystyrene (PS) or polymethyl methacrylate (PMMA) shell were elaborated. The oxide cores of about 13 nm diameter were characterized by dynamic light scattering (DLS), transmission electron microscopy (TEM), and small-angle X-ray scattering (SAXS). After the polymerization, the overall diameter reached 60 nm, as shown by small-angle neutron scattering (SANS). The behavior in solution as well as rheological properties in the molten state of the polymeric shell resemble those of star polymers. Strategies to further improve the screening of NP cores with the polymer shells are discussed.

## 1. Introduction

Hybrid inorganic–organic materials have great application potential in many technological areas [1]. The complementarity of the properties of each component allows designing materials with new potentialities and tunable properties. Numerous examples can be given in various application areas from biomedicine with superparamagnetic nanoparticles (NPs) decorated with polymers for imaging, drug delivery or targeting, or magnetic absorbents adaptable to complex geometries. The resulting material, which makes use of both the softness of the polymer shells and the specificities of the particles, constitutes an innovative way of designing new products for applications.

One main challenge consists in the elaboration of such hybrid materials with controlled shell sizes and in gram-scale batches. In this line, various strategies have been developed to attach molecules on the particle surface. Methods of direct grafting (“grafting to”) or of growth of polymer chains from a surface-attached initiator (“grafting from”) have been developed [2]. The former is easily used when the molecule is short, but the latter is preferred for thick and dense polymer coatings. The surface-initiated polymerization from an active initiator helps form a uniform surface coating of polymer chains on the surface of particles. Among the different methods, ATRP [3] permits a good control of molecular weight and polydispersity. A wide range of monomers can be used enabling the design of a wide variety of hybrid materials. As an alternative to the surface-initiated polymerization from the initiator, one should mention the methods based on the coupling of macroradicals with a radical shell attached to an inorganic core [4,5].

In the literature, several anchoring groups for ATRP initiators have been reported: for instance, carboxylic functional groups as an anchoring group for magnetic NPs [6,7]. However, this anchor is known to be unstable due to the weak interaction with the oxide surface, contrarily to silane and phosphonate leading to stronger links to iron oxides [8,9]. The phosphonate moiety is more versatile as an anchoring agent and has for instance the ability to complex metal ions through covalent bonds remaining stable at elevated temperature. Furthermore, phosphonates have a strong tendency to adsorb onto a variety of metal surfaces through the reaction with free –OH groups resulting in the formation of metal–phosphonate (M-O-P) bonds. Several studies report the ATRP polymerization on magnetite [10,11,12,13,14,15].

**Figure 1:**
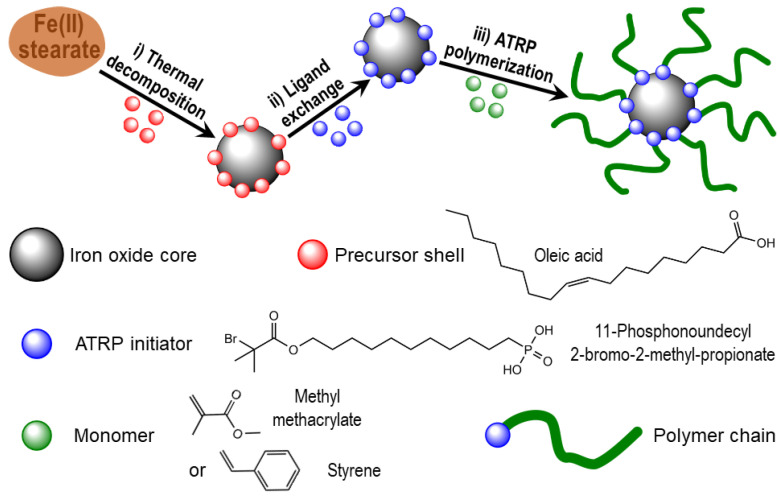
The three steps of the gram-scale synthesis of non-aggregated iron oxide/polymer core-shell nanomaterials.

In this work, core–shell iron oxide/polymer NPs were prepared by a three-step route (Figure 1). First, we prepared spherical iron oxide NPs principally composed of γ-Fe_2_O_3_ (maghemite) by thermal decomposition in the presence of oleic acid [16]. The method in the literature was improved to get tens of grams of well-controlled NPs. In a second step, an ATRP initiator was covalently grafted onto the oxide surface. As previously reported, the selected ATRP initiator contained an active tertiary bromide (initiator moiety for ATRP) and a phosphonic acid end group [17]. Then, to obtain the hybrid material, a monomer (styrene or methyl methacrylate) was polymerized from the initiator-grafted NPs. Therefore, this manuscript presents two core–shell hybrid materials: one with a maghemite core and a polystyrene (PS) shell named PS/γ-Fe_2_O_3_ and another one with the same core and a polymethyl methacrylate (PMMA) shell named PMMA/γ-Fe_2_O_3_. Iron oxide NPs, as shown by transmission electron microscopy (TEM), were well dispersed by the polymer shells. Small-angle X-ray scattering (SAXS), dynamic light scattering (DLS), and TEM were used to characterize the oxide core and combined to small-angle neutron scattering (SANS) to characterize the polymer shell and the behavior in solution of the core–shell material. The rheological properties were also studied in the molten state of the polymer shell. The features of these core–shell materials were found to mimic the behavior of star polymers.

## 2. Materials and Methods

### 2.1. Materials

Iron (II) stearate (Strem Chemicals, Bischheim, France, 9% Fe), ethanol, acetone, and hexane from Alfa Aesar were used without purification. Octyl ether (Sigma-Aldrich, Saint-Quentin-Fallavier, France, 99%) and oleic acid (90%, Alfa Aesar, Kandel, Germany) were distilled under reduced pressure. The purity of octyl ether was controlled by gas chromatography. Toluene (Carlo Erba, Val de Reuil, France, 99.9%) and anhydrous THF (Carlo Erba, Val de Reuil, France, 99.9%) were refluxed and distilled over sodium and sodium/benzophenone, respectively. Copper (I) bromide (Sigma-Aldrich, Saint-Quentin-Fallavier, France, 98%), N,N,N′N″,N″-pentamethyldiethylenetriamine (PMDETA, Sigma-Aldrich, Saint-Quentin-Fallavier, France, 99%), ethyl-2-bromoisobutyrate (Alfa Aesar, Kandel, Germany, 98%), and Anisole (Sigma-Aldrich, Saint-Quentin-Fallavier, France, 99%) were used without purification. Styrene (Sigma-Aldrich, Saint-Quentin-Fallavier, France, 99%) was distilled over sodium under reduced pressure. Methyl methacrylate (Acros Organics, Geel, Belgium) was purified by column chromatography on basic aluminum oxide (Sigma-Aldrich, Saint-Quentin-Fallavier, France). 11-Phosphonoundecyl 2-bromo-2-methyl-propionate (M_W_ = 401.28 g/mol) as an ATRP initiator with a C_11_ spacer was synthetized in the laboratory, following the experimental method reported by Maliakal et al. [18].

### 2.2. Methods of Synthesis

#### 2.2.1. NPs

First, 8 to 10 g NP batches were produced using a home-made apparatus and a thermal decomposition procedure. The installation consisted of a heating mantle, a two-liter three-necked flask, a condenser, and a mechanical stirrer. The temperature of the solution was computer-controlled (±0.1 °C accuracy). Then, 21.8 g of iron stearate (24 mM) and 19.8 g of oleic acid (70 mM) were dissolved in 315 mL of dioctyl ether by using an ultrasound bath for 30 min. To obtain a homogeneous solution, the mixture was stirred with a paddle mechanical system at 200 revolutions per minute (rpm) and heated up, first, to 110 °C for 75 min. Then, the solution was heated up to 250 °C for 1 h. Finally, the solution was heated to 288 °C with a heating rate of 5 °C/min and left at 288 °C for 24 h. The mixture was cooled down to room temperature. Ethanol was added to the solution, and the NPs were precipitated and separated via centrifugation (10 min at 14,000 rpm). The precipitate was dispersed again in chloroform. The washing process using ethanol was carried out three times. In order to select the NPs (size selective precipitation or SSP), the precipitate was dispersed in hexane, precipitated in acetone, and separated via centrifugation (8000 rpm, 10 min). The precipitate is retained, and this process was carried out three times.

#### 2.2.2. Polymer Coating

The adsorption and reaction of the ATRP initiator onto the NPs was conducted as follows. Magnetic NPs (225 mg) were mixed with an excess of ATRP initiator (84 mg, 0.209 mmol) in distilled toluene (40 mL). The solution was mechanically stirred for 72 h under argon at room temperature. After the desired time, toluene was evaporated, and the unreacted initiator was removed by three dialyses in THF (regenerated cellulose membrane of 6000–8000 Da molecular weight cutoff from Spectra/Por^®^; dialysis duration was three times 19 h). The amount of initiator adsorbed on the NP surface was evaluated by elemental analysis: Fe = 5.21 ± 0.08 mg/g and *p* = 0.117 ± 0.01 mg/g, corresponding to 42 mg (0.105 mmol) of initiator per 250 mg of magnetic NPs.

The polymerization of styrene and methyl methacrylate was carried out as follows: NPs coated with an initiator (100 mg, corresponding to 0.042 mmol of initiator), CuBr (6.0 mg, 0.042 mmol), were added to a sealed tube equipped with a magnetic bar. It was degassed (three times vacuum–argon). PMDETA (8.8 µL, 0.042 mmol, in equimolar ratio with copper) was added; then, monomers kept under argon were added: styrene (2.60 mL, 22.7 mmol, ratio to initiator 540:1) or methyl methacrylate diluted with 0.75 mL of THF (1.70 mL, 16 mmol, ratio to initiator 380:1). After several freeze–pump–thaw cycles, this tube was sealed under vacuum, put in an oil bath with magnetic stirring, and maintained at 100 °C or 35 °C (accuracy ±0.5 °C) for styrene and methyl methacrylate respectively for 24 h; the media was maintained under agitation during the time of reaction. After 24 h, the reaction mixture was diluted with THF, which was followed by precipitation in 300 mL of methanol and filtration. The hybrid material was dried in vacuum.

### 2.3. Methods of Characterization

#### 2.3.1. Powder X-ray Diffraction (PXRD)

PXRD was performed using a Siemens D8 diffractometer with Cu Kα1 radiation (λ = 1.54056 Å) and equipped with a Sol-X detector (Bruker AXS SAS, Marne La Vallée, France), in the 2θ range 27–65° with a scan step of 0.02°. The lattice parameters were refined by a method of least squares.

#### 2.3.2. Fourier Transform Infrared (FTIR) Spectroscopy

FTIR spectra were recorded using a spectrometer Digilab FTS 3000 (Bio-Rad, Marnes-la-Coquette, France). Samples were gently ground and diluted in non-absorbent KBr matrices.

#### 2.3.3. Transmission Electron Microscopy (TEM)

TEM was performed using a TOPCON model 002B (Topcon France, Mâcon, France) transmission electron microscope, operating at 200 kV, with a point-to-point resolution of 0.18 nm. The sample was suspended in THF and dispersed onto a holey carbon grid.

#### 2.3.4. Thermogravimetric Analysis (TGA)

The conversion rate and the weight fraction of polymer in core–shell NPs were determined using a SDTQ 600 apparatus (TA Instruments, Guyancourt, France) at a scanning rate of 10 °C min^−1^, under air flow, up to 1000 °C.

#### 2.3.5. Light Scattering-Size Exclusion Chromatography (LS-SEC)

The molecular weight and polydispersity (Mn and Mw/Mn) of the grafted polymer (PS or PMMA) were determined using a LS-SEC apparatus composed of a Waters chromatograph with five polystyrene gel columns (mixed B PLGel of 10 μm), a differential RID-10A refractometer (Shimadzu, Marne La Vallée, France) calibrated with polystyrene standards, a UV detector Agilent (λ = 254 et 280 nm), and a multi-angle light scattering detector (Wyatt DAWN DSP, λ = 632.8 nm). Free polymer was obtained with the following procedure: Hydrochloric acid (37%) was added to the hybrid material in solution in THF to dissolve the iron oxide. The mixture was allowed to stir overnight to obtain a free polymer solution. Then, THF was evaporated, and the polymer was extracted by decantation with chloroform, which was followed by precipitation in methanol and drying.

#### 2.3.6. Small-Angle X-ray Scattering (SAXS)

The SAXS patterns were obtained with a S-MAX3000 system equipped with a Rigaku MicroMax-007HF rotating anode (Elexience, Verrieres Le Buisson, France) for small scattering vectors (0.0065 to 0.16 Å^−1^) and with a Nanostar setup of Bruker-AXS equipped with a Fox-2D Xenocs mirror for large scattering vectors (up to 0.85 Å^−1^). In all cases, measurements were performed at room temperature on samples dissolved in THF that were contained in home-built sealed cells of 1 mm thickness and with mica windows. SAXS data were treated and set in absolute scale with 10% maximum error on the intensity calibration, using home-developed software.

#### 2.3.7. Small-Angle Neutron Scattering (SANS)

The SANS experiments were conducted on the spectrometer PACE (Laboratoire Léon Brillouin, Saclay, France) at room temperature by using 2.5 mm thick quartz cells in two configurations (λ = 4.5 Å, detector to sample distances of 4.7 and 1 m, respectively), giving access to scattering vectors between 0.01 and 0.5 Å^−1^. In all cases, measurements were performed at room temperature on samples dissolved in perdeuterated THF and DMSO. SANS data were treated and set in absolute scale with 10% maximum error on the intensity calibration, using PAsiNET software of Dr. Didier Lairez (Laboratoire Léon Brillouin, Saclay, France).

#### 2.3.8. Rheology

Shear tests, corresponding to low deformation levels (5%), were carried out in the dynamic mode in strain-controlled conditions with a plate–plate cell of an ARES spectrometer equipped with an air-pulsed oven (TA Instruments, Guyancourt, France). The samples were placed between the two plates (diameter 8 mm) fixtures at high temperature (210 °C). In dynamic mode, the frequency range was from 0.1 to 100 rad/s. We prepared samples in the form of small cylinders of 8 mm in diameter and 1.8 mm thick. For this, the powdered material (500 mg) was introduced into a mold and pressed at 50 bars. The sample is positioned on the lower plate of the rheometer, and the upper plate is lowered to a gap of 1.3 mm from the bottom tray.

#### 2.3.9. ICP-AES

Inductively coupled plasma atomic emission spectroscopy (ICP-AES) was used to determine the iron and phosphorous content of a solution obtained after the dissolution of the ATRP-coated iron oxide NPs into *Aqua regia*. The experiment was performed at the Plateforme Analytique des Inorganiques of Institut Pluridisciplinaire Hubert CURIEN, (IPHC), CNRS—Université de Strasbourg.

#### 2.3.10. DLS

Dynamic light scattering (DLS) was carried out using a Zetasizer Nano-SZ (Malvern Instruments, Palaiseau, France) on the suspension of NPs in dichloromethane.

## 3. Results

### 3.1. Elaboration of Core–Shell Iron Oxide–Polystyrene and Iron Oxide–Poly(Methyl Methacrylate)

**Figure 2:**
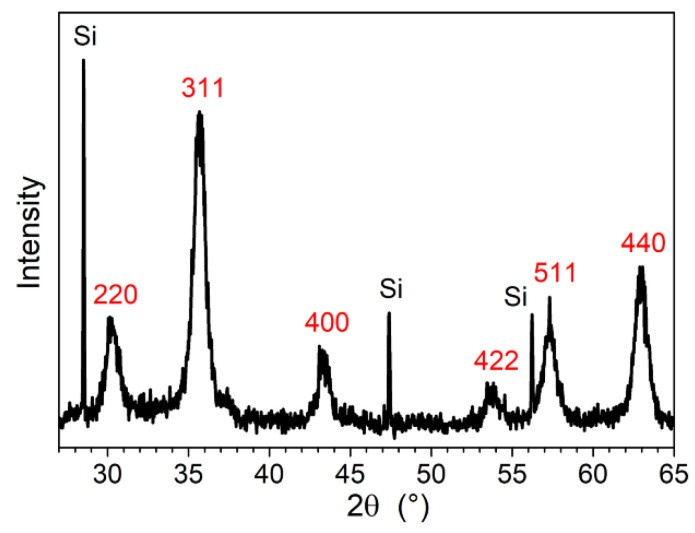
PXRD pattern of iron oxide NPs. The peaks are labeled with the hkl indices of the spinel structure. Si powder was used as a standard.

The experimental conditions of iron oxide NPs synthesis were optimized in order to obtain large batches of non-aggregated, monodisperse NPs. The best process involved obtaining a perfect dissolution of reactants at 110 °C, performing the reaction at 250 °C for 1 h, and ending the crystallization at 288 °C during 24 h. As demonstrated by PXRD, the iron oxide has the spinel structure (Figure 2). The lattice parameter, 0.8345(2) nm, is close to the maghemite one (a = 0.8351 nm ICD Data 00-039-1346).

**Figure 3:**
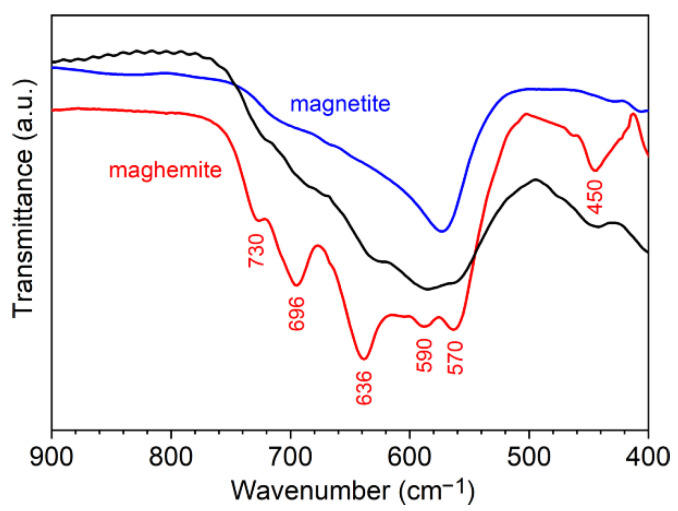
FTIR spectra of iron oxide NPs (black) compared to spectra of neat magnetite (blue) and maghemite (red) powders. Spectra with entire wavenumber range are displayed in Figure A7.

The FTIR spectrum of the NPs exhibits a broad band between those of magnetite and maghemite (Figure 3), but the bands corresponding to maghemite at 730, 696, 636, 590, 570, and 450 cm^−1^ are hardly visible in the spectrum. Accordingly, the composition and density of neat maghemite (Table A1 in Appendix B) were considered in the analysis of the core–shell structure, notwithstanding that an unknown, small amount of magnetite is likely present. In addition, hybrid material names “polymer/γ-Fe_2_O_3_” are used from here.

**Figure 4:**
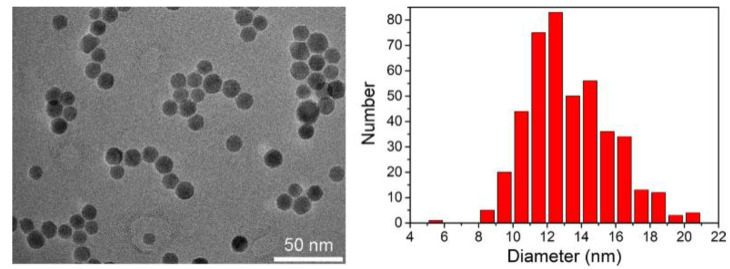
TEM image of iron oxide NPs (**left**) and size distribution measured on 436 NPs (**right**). The average diameter is 13.3 nm, and the standard deviation is 5.9 nm.

**Figure A3:**
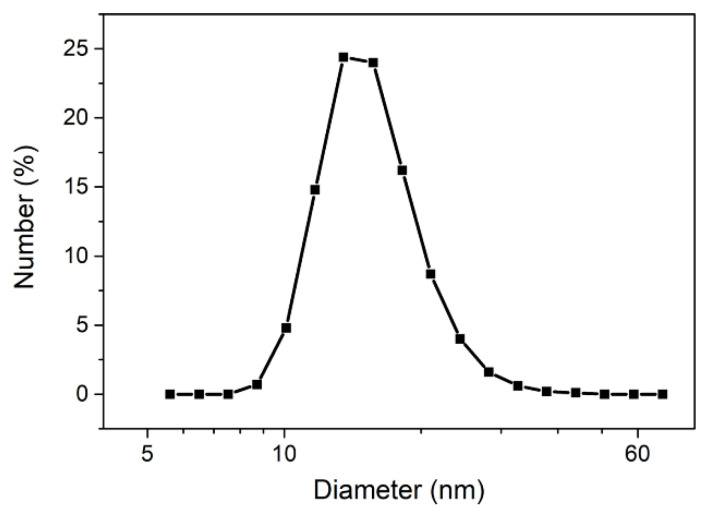
DLS curve in dichloromethane after washing and size selection.

Otherwise, the average crystallite size calculated from the PXRD peak width with the Scherrer equation, Δ(2θ) = 0.9λ/(Lcos(θ_0_)) equals 13 ± 4 nm. This value is in good agreement with the average NP diameter of 13 nm determined by TEM (Figure 4) and the diameter of 14 ± 2 nm determined by DLS (Figure A3).

**Figure 5:**
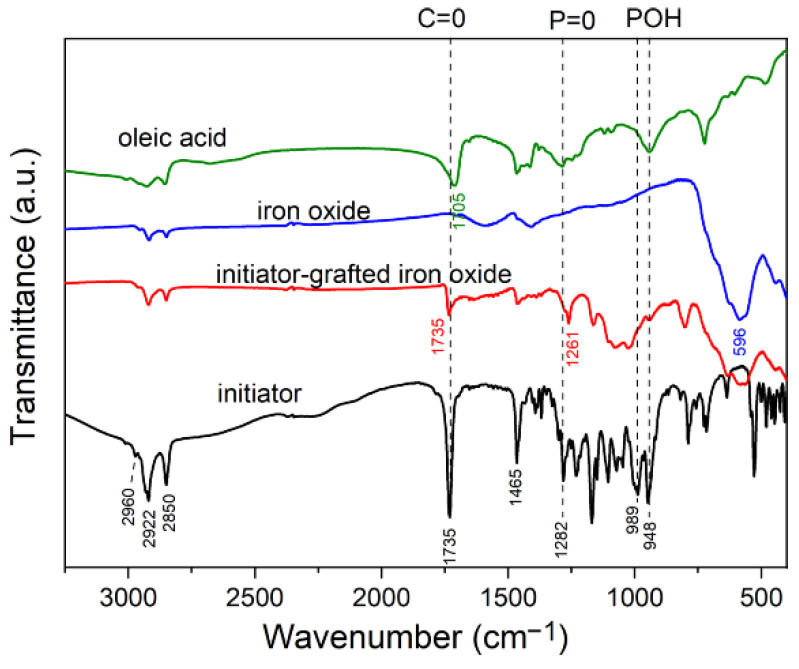
FTIR spectrum of initiator-modified iron oxide (red), compared to spectra of oleic acid (green), iron oxide (blue), and initiator (black).

The hybrid materials were made in two stages according to previous work [18]: the grafting of the initiator and the further polymerization by ATRP. The NPs synthesis expectedly led to a monomolecular oleic acid coating, which was in accordance with its low intense signature in the FTIR spectrum (Figure 5). Then, this coating was replaced by an initiator during the grafting reaction performed in toluene, and the FTIR spectrum then displayed the characteristic initiator bands. Specifically, the C=O band of oleic acid at 1705 cm^−1^ (green curve) and the P-OH bands of free initiator molecules at 989 and 948 cm^−1^ (black curve) disappear in the initiator-grafted iron oxide NP spectrum (red curve). Concomitantly, the presence of the initiator on the NP surface is attested by the appearance of the C=O band at 1735 cm^−1^ and the shift of the P=O band from 1282 to 1261 cm^−1^.

The P to Fe ratio, determined by chemical analysis (see above), and the NP average diameter from TEM gives access to the density of initiator grafting on the iron oxide surface (Appendix A). The coverage of 3.2 molecules/nm^2^ is quite dense, as the corresponding area per phosphonate group (0.31 nm^2^) is not far from the values in lamellar crystalline structures of phosphonate salts (0.22–0.23 nm^2^ in titanium (IV) diphosphonates structures CSD-YEYMIY and CSD-YEYMAQ) [19].

Two types of polymer shells have been grown from initiator coating: PS and PMMA. The FTIR spectra of the hybrid materials are shown in Figure A6. The characteristics of the polymerizations are given in Table 1. The monomer conversion of MMA is higher than that of styrene, as expected, since MMA is more reactive in ATRP reactions [3,17]. The molecular weights of the polymers obtained were found to be higher than the values expected from monomer to initiator stoichiometry by considering the monomer conversion ratio (2.5 × 10^4^ Da) (Appendix A). This is the consequence of the reduced efficiency of the initiator when grafted on NPs [20], although slowed monomer diffusion may also contribute. Indeed, the ratio of precursor and polymer coverage densities confirmed that most initiator molecules did not initiate the growing of a polymer chain. Otherwise, low polymolecularities were obtained in both cases, and these values are in accordance with those commonly reached for ATRP.

**Figure 6:**
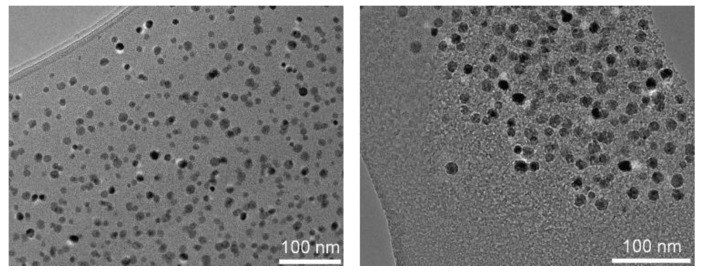
TEM images of PS/γ-Fe_2_O_3_ (**left**) and PMMA/γ-Fe_2_O_3_ (**right**).

**Figure A4:**
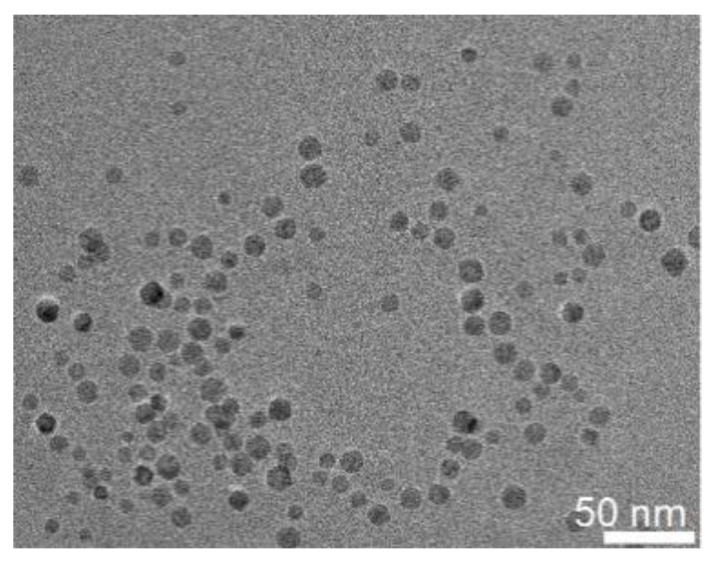
TEM images of iron oxide NPs coated with ATRP initiator molecules.

**Figure A5:**
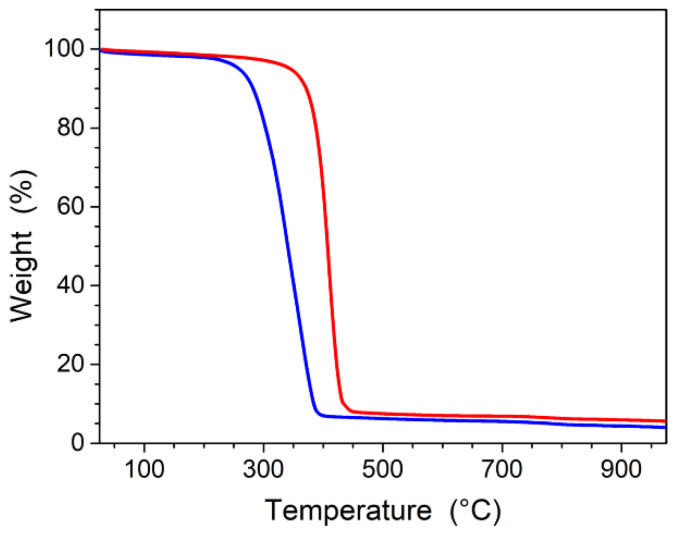
TGA of PS/γ-Fe_2_O_3_ (red) and PMMA/γ-Fe_2_O_3_ (blue). For a better accuracy, TGA experiments were combined to calcination of 20–25 mg sample amounts at 500 °C for one or several hours. Residual weight was constant after 1 h, giving an accurate iron oxide weight fraction confirming TGA results: 10.8 wt % (PS/γ-Fe_2_O_3_) and 9.5 wt % (PMMA/γ-Fe_2_O_3_).

**Figure A6:**
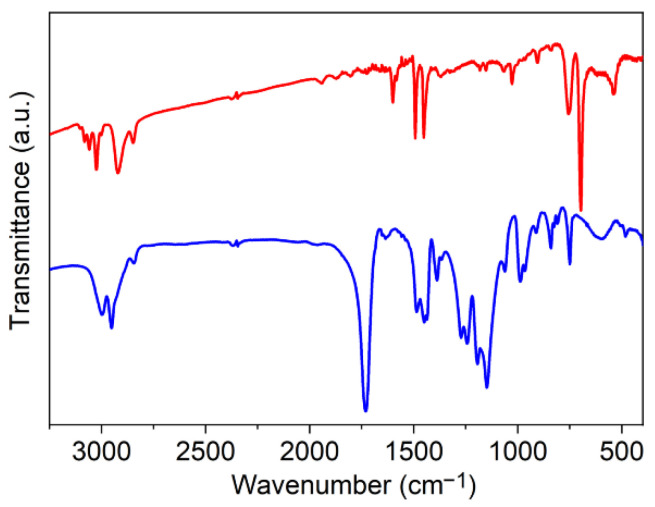
FTIR spectra of PS/γ-Fe_2_O_3_ (red) and PMMA/γ-Fe_2_O_3_ (blue) displaying the characteristic bands of the polymers.

Figure 6 shows the hybrid materials, for which NP fractions were found to be 10.8 and 9.5 wt %, respectively, as determined by TGA (Figure A5), the grafting densities being 0.46 and 1.23 polymer chains per nm^2^ (Table A2). Obviously, the NPs are non-aggregated as they stay well-separated from each other by the polymer shells. This is also the case for precursor NPs coated with oleic acid (Figure 4) and for the NPs grafted with an initiator (Figure A4).

### 3.2. Core–Shell Structure and Behavior in Solution of the Hybrid Materials

To get insight in their internal core–shell structure, the bulk hybrid materials need to be dissolved and diluted in a solvent, after which the individual core–shell objects can be probed by SAXS and SANS. The combination of these two scattering techniques is highly valuable, as they provide complementary views, i.e., high contrast of NPs with respect to polymer and solvent for the X-rays and strong contrast of the polymeric shell with respect to deuterated solvent for neutrons (Table A1 in Appendix B). In consideration of the necessity to subtract solvent scattering and other contributions from the raw scattering signal (see hereafter), the targeted solution concentrations were *ϕ_v_* = 0.02 (2% volume fraction) and *ϕ_v_* = 0.01, which could be obtained as clear solutions in THF or DMSO upon stirring the raw powder with the appropriate quantity of solvent. Unfortunately, these suspensions turned out to be metastable over several hours, after which flocculation and sedimentation became visible. This aggregation process reduced the sample concentrations effectively measured during scattering experiments. Moreover, a signal from aggregates appeared at low scattering vectors *q*. Nevertheless, the experiments were doable and provided a wealth of information.

The collected raw scattered intensities *I*(*q*) were corrected from incoherent scattering (SANS) and iron fluorescence (SAXS) and set to absolute scale. The renormalization step to single object scattering was not possible, since the real volume fractions *ϕ_v_* during experiments were not known. In SAXS, all hybrid object moieties contribute to scattering signals and lead to cross term, so that *ϕ_v_* values could not be determined from scattering curves and were left as adjustable fitting parameters. In SANS, the individual scattering length densities and volume fractions could authorize *a posteriori* estimation of the real *ϕ_v_* values using the Porod invariant, provided that density fluctuations are negligible [21]. Depending on the validity of this assumption, the products *VP*(*q*)*S*(*q*) are obtained directly or with a pre-factor close to unity, after subtraction of the solvent contribution *I_s_*(*q*). Alternatively, we obtain the same products by renormalizing scattered intensities with the contrast factor with respect to solvent Δ*ρ*^2^ = (*ρ* − *ρ_s_*)^2^, according to:

$$\times P\left(q\right)\times S\left(q\right)=\frac{I\left(q\right)-\left(1-\varphi_{V}\right)\times I_{S}\left(q\right)}{\varphi_{V}\times \Delta \rho^{2}}$$

in which *V* is the dry volume, *P*(*q*) is the form factor, such as *P*(*q* = 0) = 1, and *S*(*q*) is the structure factor. In *q*-ranges where intermolecular correlations as well as concentration fluctuations are negligible, SAXS and SANS curves were fitted with the form factor expression of successive concentric spherical shells, in which the central ball models the NPs, and the successive shells, in which different concentrations of polymer are swollen with solvent (Appendix B).

**Figure 7:**
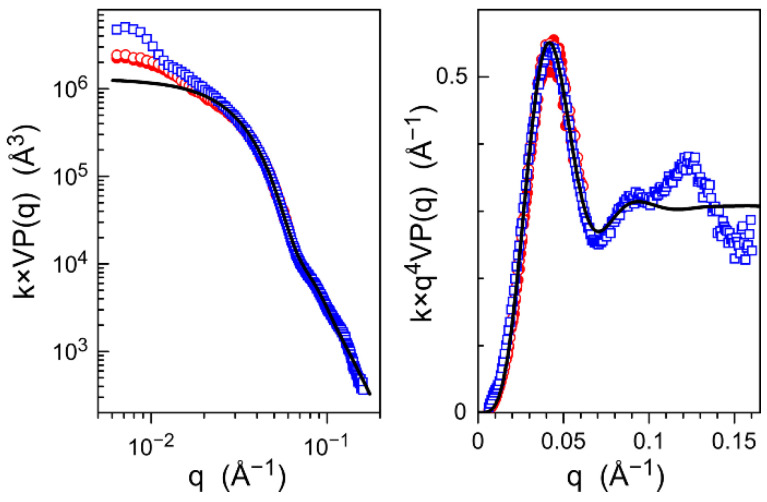
Form factor variation determined by SAXS as a function of the scattering vector (**left**) and Porod representation (**right**) of the following solutions of hybrid objects in THF: PS/γ-Fe_2_O_3_, initial volume fraction *ϕ*_v,ini_ = 0.01 (red solid circles, curve stops at 0.06 Å^−1^); PS/γ-Fe_2_O_3_, *ϕ*_v,ini_ = 0.02 (red open circles, curve stops at 0.06 Å^−1^); PMMA/γ-Fe_2_O_3_, *ϕ*_v,ini_ = 0.01 (blue solid squares); PMMA/γ-Fe_2_O_3_, *ϕ*_v,ini_ = 0.02 (blue open squares); the black line is the fit for a Gaussian distribution of balls, with 13 nm mean diameter and 6 nm standard deviation.

In SAXS curves, *VP*(*q*)*S*(*q*) does not depend on particle volume fraction for *q* > 0.02 Å^−1^, indicating that interactions between objects can be neglected and thus that the structure factors are close to unity in this *q*-range (Figure 7). Given the high electronic density and the size range of the inorganic cores compared to the organic shell and solvent, the variation of *VP*(*q*) in the intermediate *q* range reflects the decrease in the core contribution, while the shell merely interferes in contrast and background. A characteristic *q*^−4^ power law with superimposed damped oscillations is followed and reflects the spherical shape and the relatively small polydispersity of the cores. From logarithmic and Porod representations, a fit with a Gaussian distribution of homogeneous balls gives a mean diameter of 13 nm for a standard deviation of roughly 6 nm, which is consistent with values from TEM (Table A2 in Appendix B). However, the matching of the model is poor at large *q*, which is presumably because of the multi-modal size distribution. Deviation is also observed at small *q*-values, roughly below *q* = 0.02 Å^−1^, corresponding to the Guinier range associated with cores. Then, the contribution of the cores to the SAXS signal only shows a residual variation toward *q* = 0, and therefore, the further intensity increase mainly originates from entire objects, given the low but non-negligible electron density contrast of the shell with respect to solvent and its high volume fraction (Appendix B). The contribution of intermolecular correlation can be neglected, since the deviation was equivalent for diluted and concentrated solution and also in proportion to the contrast between the polymer shell and solvent (higher for PMMA).

On the contrary, the NPs show very low contrast with respect to deuterated solvent in SANS (and even almost no contrast in the case of THF-d8), while they represent a small fraction of the hybrid object volume. Hence, the contribution of the cores to the overall scattering is either almost completely negligible, as THF-d8 is the solvent, or low, as DMSO-d6 is the solvent. In both cases, the hybrid objects can be seen as “hairy balls”, i.e., with a core having a constant scattering length density and a concentric shell in which the scattering length density depends on the distance from the core, provided that the concentration fluctuations are neglected.

In view of the polymer chains radiating from the hidden NP cores, the hybrid objects mimic star-like polymers, except that a cavity of a certain volume replaces the branch points and that the mean number of branches *f* is particularly large: *f* = 240 for PS/γ-Fe_2_O_3_ and *f* = 650 for PMMA/γ-Fe_2_O_3_ (Table A2). Given the good solubility of PS and PMMA in THF, the shell is for sure strongly solvated, and thus, the system can be pertinently described using the approach developed by Daoud and Cotton for star polymers with a large number of branches [22,23]. According to this model, the core and the internal part of the polymer shell behave as a unique swollen ball with a radial solvent concentration profile, while in the outer shell, solvated branches are viewed individually and distributed within spherical volumes around segments (i.e., blobs). Consequently, the scattering curve merely reproduces the contribution of the large-size inhomogeneous balls at small and intermediate *q*, which is relayed at high *q* by a regime with a predominant contribution of blobs, whose largest size ξ(R) sets the cross-over zone (here, R is the radius of the ball describing one hybrid particle).

In order to appreciate the relevance of this view, theoretical scattering curves were calculated for representative populations of isolated inhomogeneous balls. In accordance with SAXS, TEM, and chemical composition, the mean diameters of the dry shell and the oxide NP were set to respectively 60 and 13 nm for both hybrids. To account for shell size variation, a lognormal distribution of polydispersity of 1.4 was tentatively considered. The incorporated solvent was assumed to follow the radial profile of the original Daoud–Cotton model, consisting of a core region free of solvent beyond which the concentration decreases according to the r^−1^ power law for the Gaussian case. Therefore, the location of the boundary between the core and the solvated shell determines the degree of solvation and remains the fitting parameter of the model. The best adjustment between calculated curves and experimental points was realized for the steep decrease in the intermediate *q*-range dominated by the form factor variation of the swollen balls.

**Figure 8:**
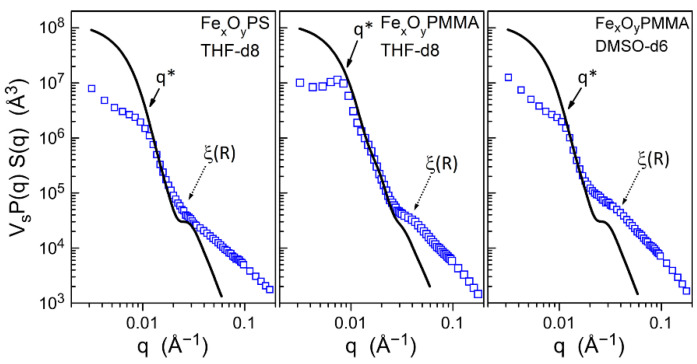
SANS curves determined as a function of scattering vector for PS/γ-Fe_2_O_3_ in THF-d8 ((**left**); *ϕ*_v_ = 0.0058), PMMA/γ-Fe_2_O_3_ in THF-d8 ((**middle**); *ϕ*_v_ = 0.0079), and PMMA/γ-Fe_2_O_3_ in DMSO-d6 ((**right**); *ϕ*_v_ = 0.0047); the black lines are the theoretical scattering curve for isolated hybrid objects swollen with solvent; dotted arrows: predicted cross-over zone ξ(R) to predominant blob scattering of isolated objects; solid arrows: cross-over zone q* towards *q*-range of interacting objects.

Optimal fits were obtained for the high degrees of solvation of the shell (defined as the volume fraction of solvent in swollen objects X_vs_ = 93%, 83%, and 92% for the curves in Figure 8). Unfortunately, these values are poorly reliable in consideration of unknown experimental parameters and rough approximations, but they confirm the expectations based on the composition of dry hybrid objects. The statistical NP surface covered by a single chain is indeed as large as 2.2 nm^2^ for PS/γ-Fe_2_O_3_ and 0.81 nm^2^ for PMMA/γ-Fe_2_O_3_; i.e., it is substantially above the cross-sections of PS and PMMA chains (0.65 and 0.55 nm^2^) (Table A2), and it leaves enough space to soak the polymer shell with solvent.

Whatever the real solvation degrees, the calculated curves clearly deviate from the experimental points at high *q*-values and confirm the cross-over to the regime of predominant blob scattering. Another deviation is observed at small *q* and reveals the setting on of interactions between swollen balls. The cross-over zone, located around q* ≈ 0.008–0.01 Å^−1^, corresponds to distances 2π/q* (≈60–80 nm), which are even below the entire swollen object diameter (100–150 nm, deduced from fitted X_vs_ values). Consequently, solutions contain aggregates whose internal structure could comply with a peripheral continuum of intermingled polymer blobs separating swollen balls, as proposed for the semi-dilute regime in the frame of the Daoud–Cotton model [22,23]. Apart from the interactions between neighboring balls, the blob size distribution is modified with respect to isolated objects, which in turn affects the cross-over zone and the scattered intensity profile. However, experiments are insufficient to further discriminate behaviors in the presence of flocculation.

### 3.3. Rheological Behavior

**Figure 9:**
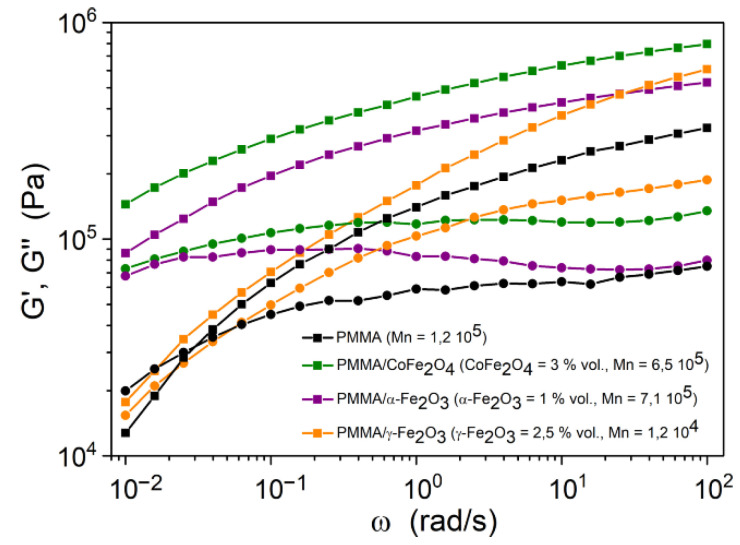
Variation of the storage G′ (■) and loss G″ (●) moduli of pure PMMA and of PMMA chains grafted onto the surface of NPs (PMMA/NPs), as a function of frequency ω at a temperature of 210 °C.

In Figure 9, the storage G′ and loss G″ moduli of PMMA/γ-Fe_2_O_3_ core–shell NPs are compared to those of pure PMMA and to aggregated hybrid materials PMMA/α-Fe_2_O_3_ (hematite) and PMMA/CoFe_2_O_4_ (cobalt ferrite) whose syntheses were published elsewhere [15].

The pure polymer shows a classical behavior with a rubbery plateau and the beginning of the terminal flow zone at frequencies below 0.03 rad/s. A characteristic relaxation time can be defined as the inverse of the frequency corresponding to the intersection of the G′ and G″ curves. Its value is close to 30 s for pure PMMA.

The polymers grafted to the surface of the NPs have much higher molar masses than the pure polymer. For these samples prepared by the “grafting from” method, we find that the elastic character (G′) is predominant with respect to the viscous character (G″), even for small frequencies. Therefore, we do not observe any flow zone in the experimental frequency range, and therefore, the relaxation time should be larger than 100 s. Although samples PMMA/α-Fe_2_O_3_ and PMMA/CoFe_2_O_4_ exhibit a nearly solid (rubbery) behavior in the whole frequency range, which is in agreement with the high molar mass of grafted chains, the variation of the moduli is quite different in the particular case of PMMA/γ-Fe_2_O_3_. At low frequencies, the moduli of this sample decrease rapidly with decreasing frequency, which is in agreement with the smaller molar weight of the grafted chains, but no terminal flow zone and no crossing of the G′ and G″ curves is observed. The behavior at low frequencies is close to a power-law, and it is analogue to those reported for polymers having a star morphology [24,25].

## 4. Discussion

The detailed characterization of the hybrid materials definitively validates the proposed “three-step route” for the preparation of large-scale batches of spherical NPs, which are homogenously separated from each other by covalently bound polymer shells. After the initial synthesis of the precursor-coated iron oxide NPs, the precursor ligands were quantitatively exchanged by a dense coverage of chemically bound polymerization initiator molecules. The last step consisting of the reaction of the initiator with the monomer and the growing of radiating polymers chains was less efficient, since only 14% and 38% of bound molecules initiated a chain, depending on the polymer nature, which is a finding that is line with the literature and explainable by the lower accessibility of molecules bound to the surface [20,26]. However, the moderate density of the polymer chain coverage was compensated by increased chain lengths, which allowed reaching a thick overall polymer embedding. Thereby, PMMA and PS showed different monomer reactivity and grafting density, but the final hybrid objects showed comparable dry diameters about 60 nm, extending the 13 nm average diameter of oxide cores.

The preparation of hybrid material solutions and their investigation by SAXS/SANS combination confirmed these dimensions and further validated the persistence of the polymer coverage after dispersion in solution. The experimental curves presented cross-overs between three scattering regimes, recalling those observed for star polymers with a large number of branches. These two systems indeed resemble polymeric architectures that associate a dense core constituted by NP or by crowded chains and a corona of radiating chains with a surface per chain expanding with distance to core. Intuitively, these coronas should be soaked by solvent in a similar way and with similar interactions between solvated chains. Going on this analogy, the star polymer approach developed by Daoud and Cotton was applied to hybrid materials for the analysis of the scattering range beyond the first cross-over that probes the internal structure of the solvated polymer shell. Effectively, the shape of the experimental curves could be reproduced considering an intermediate q regime dominated by the scattering of the internal shell in which the polymer behaves as a unique swollen ball and a cross-over to a wide-q regime dominated by the scattering of the outer shell constituted of individually viewed solvated branches. However, uncertainties about real concentrations and size distributions, as well as the missing single object low-q regime, precluded a more detailed analysis and the definitive validation of this approach.

The analogy with star polymers is not applicable to the range below the first cross-over describing the behavior of entire objects. This is particularly true since interactions between entire objects are present and result in the progressive flocculation of solutions during aging. Remarkably, this dynamical process occurs in solutions which are one order of magnitude more diluted than the calculated overlap concentration *ϕ*_v_* of the isolated swollen objects, while interactions between polymers in good solvent conditions are repulsive, even for stars with a large number of arms [27,28]. Therefore, the aggregation comes from the insufficiently screened NPs and might ultimately be suppressed with a denser coverage of polymer chains. The effect of the polymer chain length is hardly predictable: on the one hand, longer chains are unfavorable to aggregation through the increased polymer volume fraction, and thus, the increased NP spacing and the reduced density difference with solvent. On the other hand, longer chains intermingle more easily and form longer spacing, and this might stabilize the formation of a continuum of solvated chains and eventually favor the aggregation. Finally, reducing the NP size will of course delay the aggregation.

## 5. Conclusions

In this work, core–shell PS/γ-Fe_2_O_3_ and PMMA/γ-Fe_2_O_3_ have been prepared using an original “three-step route” characterized by core and shell composition and studied regarding behavior in bulk and in solution by using a broad combination of techniques.

The developed “three-step route” turned out to be an efficient way to prepare large-scale batches of hybrid materials, and it potentially allows varying the core–shell dimensions upon the variation of polymerization conditions. However, the versatility of the method is limited by the altered reactivity of the initiator molecules bound to the NP surface with respect to classical ARTP performed in solution. There are different leads to overcome these limitations. Here, the initiator group was connected to anchor through a C_11_ spacer, but its accessibility will surely be changed with a longer or a shorter spacer. Then, the surface polymerization was only applied to MMA and styrene, but there are plenty of alternative monomers showing different reactivities and giving access to other reaction conditions. Moreover, the use of other monomers paves the way for the design of hybrid materials with different properties in bulk and in solution.

The hybrid materials showed behaviors in the molten state of the polymer and in solution that largely comply with those of star polymers, meaning that the properties of the polymer embedding effectively prevail over those of NPs. On the other hand, the screening of the NPs was not sufficient to avoid flocculation with aging of the solutions.

As a matter of fact, propensity to aggregate is related to NP size, which is confirmed by the lower tendency of other core–shell materials with substantially smaller NPs to aggregate [29]. Therefore, one strategy to get rid of aggregation will be to reduce the NP size by maintaining the same polymer grafting. For NPs of the same and larger size, the only way will probably be to develop denser polymer coverages along with the strategies outlined above. Hence, the topic has room for improvements and merits to concentrate more efforts. Once stability in solution in achieved, deeper fundamental studies on the internal structure of objects might be conducted and possible material applications envisaged.

## Figures and Tables

**Table 1:** Characteristics of the ATRP polymerizations (see Appendix A for details).

Hybrid Material	Converted Monomer (%)	Mn (Da)	Mw/Mn	Initiator Efficiency (%)
PS/γ-Fe_2_O_3_	45	30 × 10^4^	1.23	14
PMMA/γ-Fe_2_O_3_	62	12 × 10^4^	1.52	38

**Figure A1:**
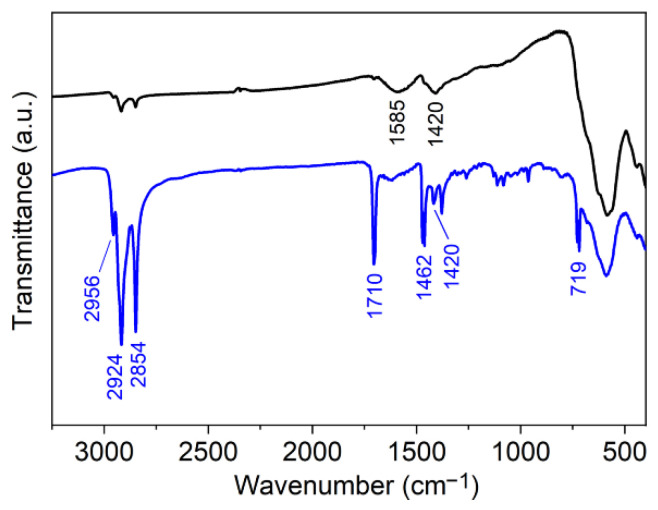
FTIR spectra before (blue) and after (black) washing and size selection.

**Figure A2:**
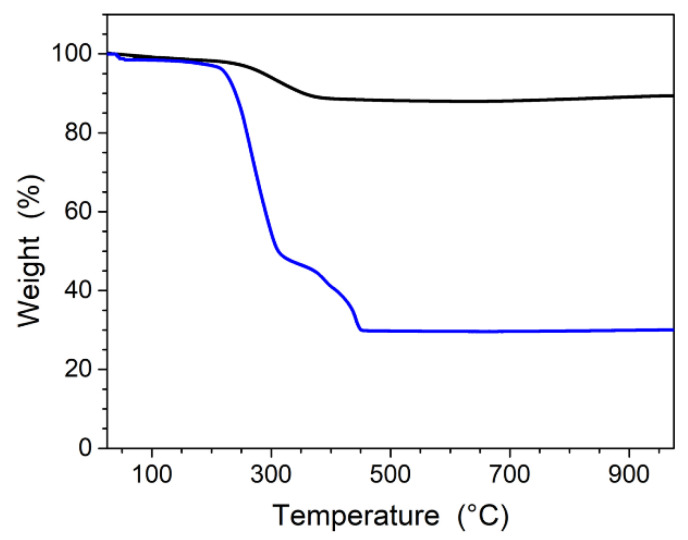
TGA curves of before (blue) and after (black) washing and size selection.

**Figure A7:**
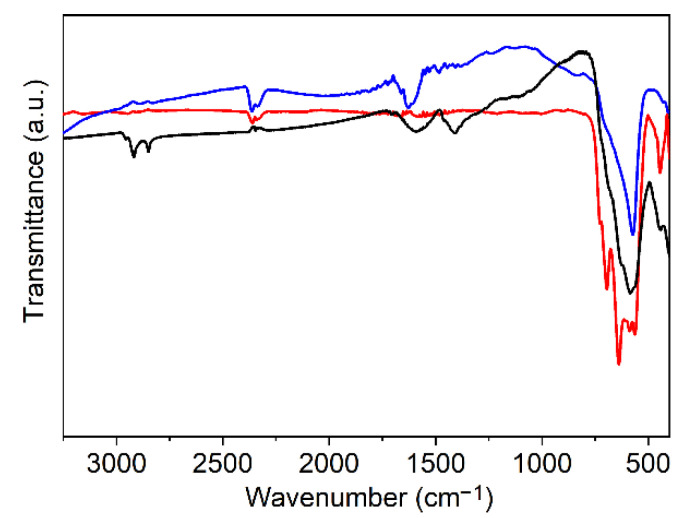
FTIR spectra with entire wavenumber range of iron oxide NPs (black) compared to spectra of neat magnetite (blue) and maghemite (red) powders. An expansion of spectra to wavenumber region of interest (900 to 400 cm^−1^) is shown in Figure 3.

## Data Availability

Not applicable.

## Appendix A. Characteristics of Three-Step Route Synthesis

### Appendix A.1. Synthesis of Iron Oxide NPs

For the large-scale synthesis, a home-made apparatus was used. Installation consisted of a heating mantle, a two-liter three-necked flask, a condenser, and a mechanical stirrer. The temperature of the solution was controlled by a computer via a proportional–integral–derivative (PID) controller. The advantage of this home-made installation is that it allows varying easily the amounts of reagents by an easy adaptation of the needed heating power. In the present case, the quantities of reactants were as follows: 21.8 g of iron stearate (24 mM) and 19.8 g of oleic acid (70 mM) dissolved in 315 mL of dioctyl ether by using an ultrasound bath for 30 min. To obtain a homogeneous solution, the mixture was heated at 110 °C for 75 min and stirred with a paddle mechanical system at 200 revolutions per minute (rpm). The temperature was increased to 250 °C and maintained during variable times, typically 3 h as for the batch of Figure 2, Figure 3, Figure A1 and Figure A2. Finally, the temperature was increased to 288 °C and maintained for 24 h. The washing of NPs was carried out according to the reference synthesis, dividing the solution into 15 batches. This procedure allowed the synthesis in large amounts of non-aggregated nanoparticles covered by oleic acid (8.6 g for the batch selected for these figures).

These NPs were effectively coated with oleic acid, as shown by FTIR spectroscopy (Figure A1). The spectrum exhibits the broad band between 500 and 700 cm^−1^ that is characteristic of iron oxide and strong bands that are characteristic of the organic residue. After washing and size selection, the absorption bands of ungrafted oleic acid molecules at 1710 cm^−1^ and of iron stearate at 719 cm^−1^ are not present. However, the band at 1420 cm^−1^ assigned to -COO groups is still visible, meaning that some oleic acid molecules are bound to the surface of nanoparticles. Consistently, the weight loss due to the combustion of the oleic acid shell during TGA experiments drops from 69.5% to 10.5% after washing and size selection (Figure A2).

### Appendix A.2. Grafting of Initiator on Iron Oxide Surface

The average NP surface covered by an initiator molecule *S_Ini_* can be calculated from the diameter of the spherical NPs and from the phosphorous over iron content.

(A1) $$S_{Ini}=\pi \times {D_{C}}^{2}\times {\left[n_{Ini,NP}\right]}^{-1}=\pi \times {D_{C}}^{2}\times {\left[\frac{\pi /6\times {D_{C}}^{3}}{V\left[Fe_{2}O_{3}\right]}\times 2\times \frac{\left[P\right]}{\left[Fe\right]}\right]}^{-1}$$

in which *Dc* is the NP diameter obtained from TEM and SAXS (Appendix B), *n_Ini,NP_* is the average number of initiator molecules per NP, *V*[*Fe*_2_*O*_3_] is the volume of a Fe_2_O_3_ unit (Table A1), and [*P*]/[*Fe*] = 0.0405 ± 0.0041 is the phosphorous over iron molar ratio in the hybrid material determined by ICP-AES (see above in Section 2.2.2).

### Appendix A.3. Growing of the Polymer Shells

From TGA and LS-SEC, it is possible to determine the monomer conversion rate, the experimental and calculated molecular weights of the polymer, and the efficiency of the initiator. Details of the calculations are given below.

The monomer conversion (*MC*) represents the monomer fraction that was effectively converted to polymer. It is obtained from the weight fraction of the organic shell in the final hybrid material and the initially engaged monomer quantity.

(A2) $$MC\;\left(\%\right)=100\times \frac{m_{NP}\times X_{Pol}}{m_{Mon}}$$

in which *m_Mon_* and *m_NP_* are the monomer and hybrid material masses, and *X_Pol_* = is the weight fraction of the polymer, as obtained from weight loss in TGA (Figure A5).

The molecular weights *M_n_* and polymolecularities of the polymer chains were determined using LS-SEC. The calculated molar mass *M_n_*_,*cal*_ represents the molecular weight that would be obtained if all initiator molecules had initiated the growing of a polymer chain. This value is calculated for the monomer fraction that was effectively converted to polymer.

(A3) $$M_{n,cal}=\frac{{\left[Mon\right]}_{0}}{{\left[Ini\right]}_{0}}\times M_{w,Mon}\times MC$$

in which [*Mon*]_0_/[*Ini*]_0_ is the molar ratio of the monomer and initiator in the reaction mixture, *M_w_*_,*Mon*_ is the molar mass of the monomer, and MC is the monomer conversion determined before.

## Appendix B. Information and Data from the Combined SAXS and SANS Study

A combination of SAXS and SANS varies the contrast of iron oxide core and polymer with respect to solvent, which allows probing the core–shell structure. Parameters for contrast and volume fraction calculations are listed in Table A1.

**Table A1:** Characteristics of the inorganic phases constituting the nanoparticles, the repeat units of polymer shells (r.u.), and the solvents of scattering experiments.

Molecular Unit	ρ ^1^ (g/cm^3^)	V ^2^ (nm^3^)	ρ_e_ ^3^ (10^10^ cm^−2^)	ρ_coh_ ^4^ (10^10^ cm^−2^)
γ-Fe_2_O_3_	4.860	0.0546	39.3	6.65
PMMA r.u.	1.19	0.140	10.8	1.06
PS r.u.	1.04	0.166	9.59	1.41
THF	0.889	0.1347	8.37	0.18
THF-d8	0.985	0.1352	8.35	6.34
DMSO-d6	1.184	0.1178	10.05	5.26

^1^ ρ: density; ^2^ V: molecular volume; ^3^ ρ_e_: electronic density; ^4^ ρ_coh_: neutron coherent scattering length density.

The experimental curves display three scattering regimes separated by cross-overs *q** and ξ(R). The contribution of interactions between entire objects in the scattering range below *q** precludes its quantitative exploitation. The range above ξ(R) is dominated by individual chain scattering and was not investigated further. In the range between these two cross-overs, interactions are negligible, and the polymer shell is probed as a unique swollen ball. Therefore, the scattering intensity variation in this range gives access to core and dry shell dimensions and insight in the solvent distribution in the swollen shell.

The analytical approach exploits the analogy with a well-studied reference system, namely star polymers for which the polymer branches radiate around a congested central core, similarly to the polymer chains grafted on the NP surface [22,23]. Therefore, we hypothesized that the radial concentration profile demonstrated for the swelling of star polymers also applies to the swelling of the NP shells. Specifically, this profile includes two radii r_in_ and r_out_ separating regions in which the decrease in concentration with radius r follows different laws: an inner region of dry polymer, an intermediate shell with a decrease according to an r^−1^ power law and an outer diffuse shell with an r^−4/3^ power law. Both radii were taken in account in our first simulation trials, but it appeared that calculated curves were nearly insensitive to the position of r_out_. This transition radius was ignored, and the r^−1^ power law was applied to the whole object in the next simulations. As a result of these simplifications, three parameters are enough to define the individual swollen object: the NP diameter D_C_, the entire object dry diameter D_CSh_, and the volume fraction of solvent X_vs_ determined by the position of r_in_.

Another feature necessary for simulation is the polydispersity of the objects. To account for the size distribution of the NPs, a population of NPs of the same standard deviation as experimental NP batches measured with TEM was created, and the position of the average diameter was optimized by fitting SAXS curves. It was more complicated to account for the distribution of shell sizes. The only data from experience are the polymolecularity of the polymer measured after dissolution of the NP cores. However, this type of polydispersity is in part compensated by the fact that polymer shells associate numerous chains, whereas nothing is known about other contributions such as the variation of overall polymer chain coverage between NPs. To parametrize our calculations, we tentatively considered a lognormal distribution of shell sizes with standard deviation 0.25, which would correspond to a polymer of polymolecularity 1.4. Finally, it was considered that the inner dry polymer shell had the same diameter 2r_in_ for the whole population of objects. Then, the position of r_in_ (and thus X_vs_) was optimized to best fit SANS (and SAXS) curves.

To calculate the scattering contribution of individual objects, the swollen polymer region was divided into hundreds of discrete concentric shells in which the polymer concentration is constant. Then, the scattered intensity was calculated using the theoretical form factor expression for concentric shells [30].

(A4) $$P\left(q\right)={\left[\frac{3}{\sum^{\;}\left(\left(\rho_{j-1}-\rho_{j}\right)\times V_{j}\right)}\times \overset{N}{\underset{i=1}{\sum }}\left(\left(\rho_{i-1}-\rho_{i}\right)\times V_{i}\times \frac{\sin\left(qR_{i}\right)-\left(qR_{i}\right)\cos\left(qR_{i}\right)}{{\left(qR_{i}\right)}^{3}}\right)\right]}^{2}$$

in which *ρ_i_* is the scattering length density of NP or of concentric shells calculated from the concentration profile, *ρ*_0_ is the solvent, and *V_i_* is the sphere volume of radius *R_i_*. The same procedure and object populations were used for SAXS and SANS curve fitting after modification of the scattering length densities according to Table 1.

It is clear that this approach requires further validation and new developments supported by other sets of experimental data. However, the present computing already gives access to object dimensions in solution and evaluates the degree of soaking of the polymer shell with solvent. Results are gathered in Table A2.

**Table A2:** Characteristics of the hybrid materials in dry and swollen state.

Hybrid Material	Mn ^1^ (g/mol)	V_Pol_ ^3^ (nm^3^)	D_C_ ^5^ (nm)	V_Sh_ ^7^ (nm^3^)	*f* ^9^	*f*/*f*_max_ ^11^ (%)
	ρ_Pol_ ^2^ (g/mol)	σ_Pol_ ^4^ (nm^2^)	D_CSh_ ^6^ (nm)	S_C_ ^8^ (nm^2^)	S_Pol_ ^10^ (nm^2^)	X_vs_ ^12^ (%)
PS/γ-Fe_2_O_3_	297 × 10^3^	474	13	116 × 10^3^	244	30
	1.04	0.655	60.6	531	2.18	93
PMMA/γ-Fe_2_O_3_	120 × 10^3^	167	13	109 × 10^3^	653	68
	1.19	0.55	59.5	531	0.81	83 {92}

^1^ Mn: number average molar mass of polymer chains from LS-SEC (Table 1); ^2^ ρ_Pol_: dry polymer density; ^3^ V_Pol_ = Mn/6.022 × 10^23^/ρ_Pol_ × 10^21^: average polymer volume; ^4^ σ_Pol_ = V_r.u._/L_r.u._: cross-sectional area of a polymer chain, V_r.u._ being the repeat unit volume (Table A1) and L_r.u._ = 0.254 nm being the repeat unit length; ^5^ D_C_: average NP diameter, as optimized from scattering curve fitting; ^6^ D_CSh_: average dry diameter of core–shell objects, as optimized from scattering curve fitting; ^7^ V_Sh_ = (π/6) × (D_CSh_^3^ − D_C_^3^): average shell volume; ^8^ S_C_ = π × D_C_^2^: average NP surface area; ^9^ *f* = V_Sh_/V_Pol_: average number of chains grafted on the NP surface; ^10^ S_Pol_ = S_C_/*f*: NP surface area per chain; ^11^ *f*/*f*_max_ = 100 × σ_Pol_ / S_Pol_: coverage ratio of NP surface by polymer chains; ^12^ X_vs_: volume fraction of solvent in swollen objects from scattering curve fitting, the value between brackets being for DMSO and the two other values for THF; the corresponding dry polymer inner shells diameters are respectively 2r_in_ = 26 nm (PS), 34 nm (PMMA, THF), and 27 nm (PMMA, DMSO).
